# Supplementary figures and images for: The MITF paralog tfec is required in neural crest development for fate specification of the iridophore lineage from a multipotent pigment cell progenitor
Source: PLoS One. 2021 Jan 13;16(1):e0244794. doi: 10.1371/journal.pone.0244794 (PMC7806166; doi:10.1371/journal.pone.0244794)

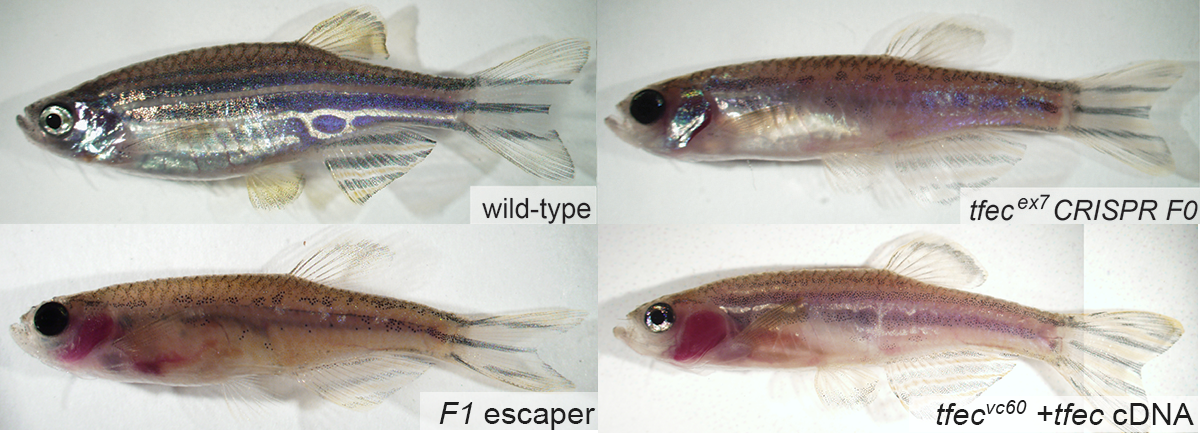

Supplement: S1 Fig — Compared to WT adult (A), G0 tfec crispant (mosaic) adult shows patches of iridophore loss on eye and flank (B). When two tfec CRISPR G0 founder fish were mated, almost all of the offspring lacking iridophores died as larvae, but one escaper survived to adulthood and the absence of iridophores persisted (C). Of the homozygous tfecvc60 embryos injected with a Tol2 transposon containing the tfec promoter and cDNA and Tol2 transposase mRNA, one survived to adulthood and displayed partial rescue of adult iridophore pigmentation (D). Scale bar: A-D: 0.5 cm; D: 0.35 cm. (TIF) [file pone.0244794.s001.tif]

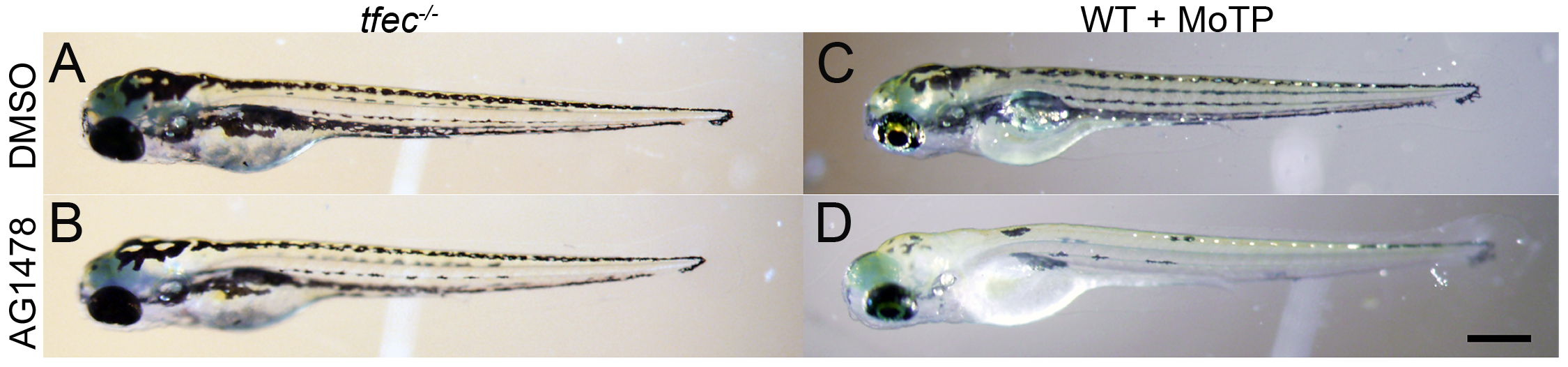

Supplement: S2 Fig — (A,B) tfec homozygous mutant larvae treated with AG1478 (B) recover melanocytes to levels of DMSO-treated larvae (A) by 4.5 dpf. (C,D) Wild-type larvae, treated with MoTP reagent at an early stage to ablate melanocytes, and with AG1478 (D) show strongly reduced melanocyte numbers by 4.5 dpf, compared to MoTP and DMSO-treated siblings (C). Scale bar: 250 μm. (TIF) [file pone.0244794.s002.tif]

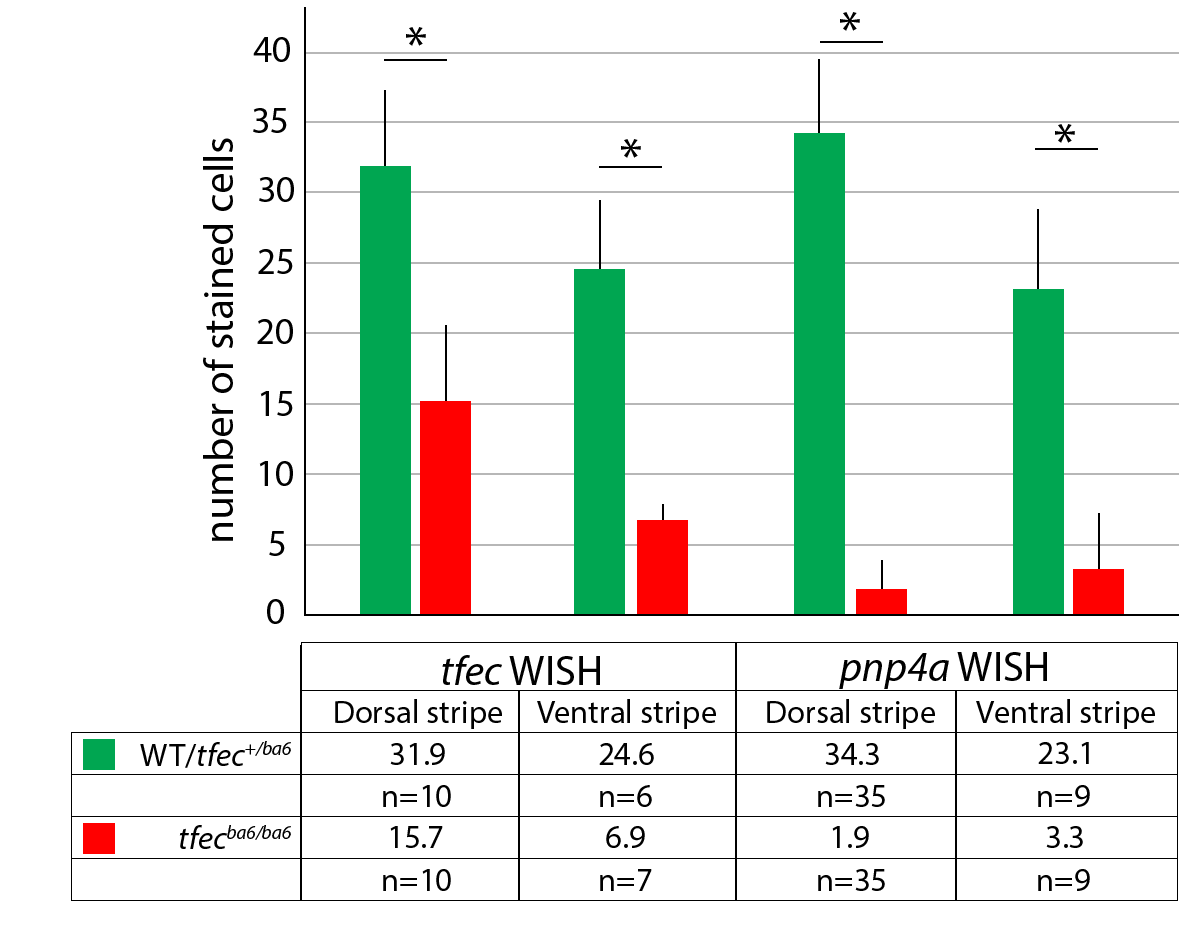

Supplement: S3 Fig — Positive cells were scored along the dorsal and the ventral stripe of known tfecba6/ba6 embryos (red bars) and of wild-type or heterozygous siblings (green bars). *: p-value < 10−3. (TIF) [file pone.0244794.s003.tif]

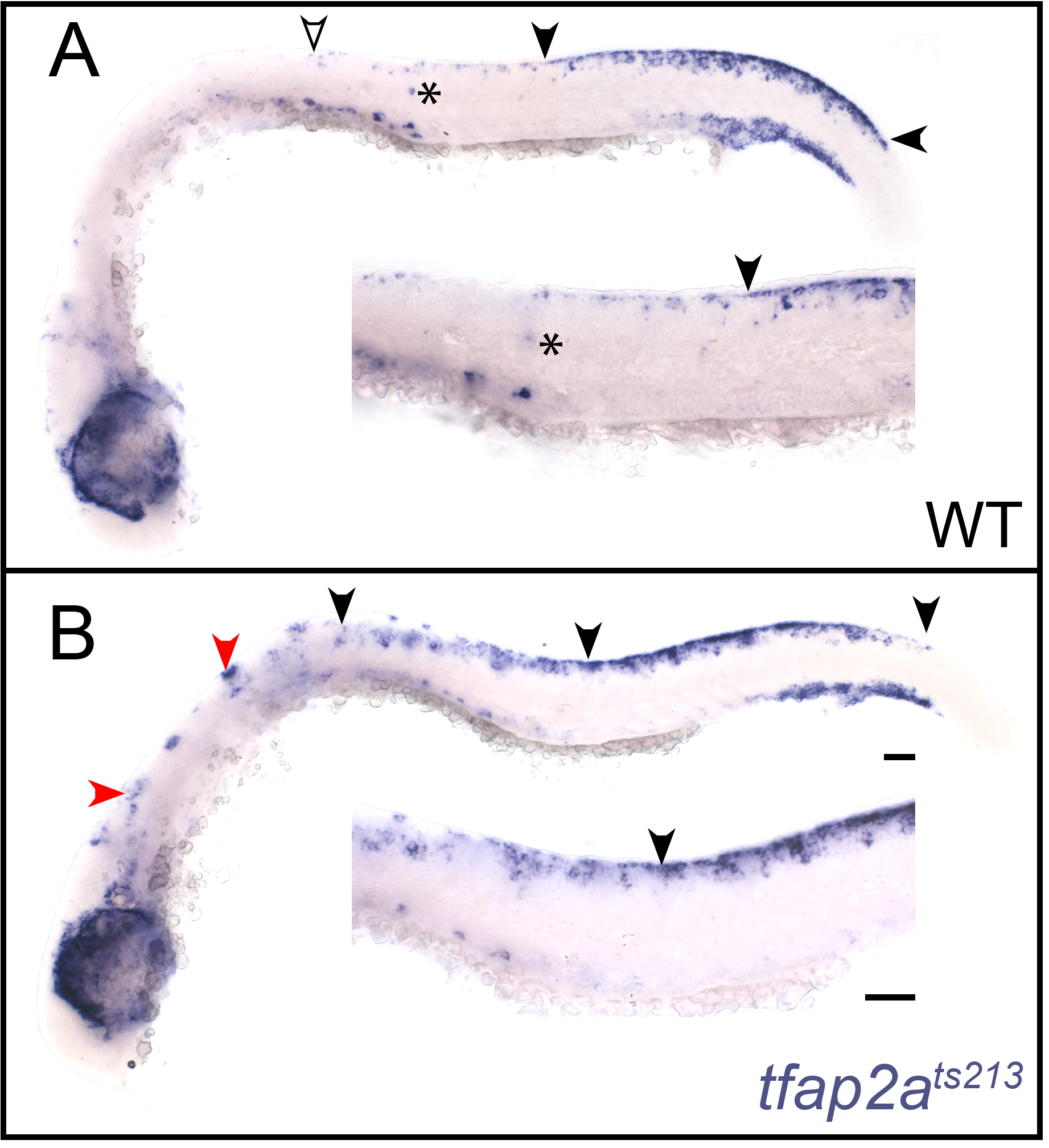

Supplement: S4 Fig — (TIF) [file pone.0244794.s004.tif]
