# Supplementary material for: The MITF paralog tfec is required in neural crest development for fate specification of the iridophore lineage from a multipotent pigment cell progenitor
Source: PLoS One. 2021 Jan 13;16(1):e0244794. doi: 10.1371/journal.pone.0244794 (PMC7806166; doi:10.1371/journal.pone.0244794)
Supplement: S1 Table — For melanocyte counts at 4 dpf, to derive the average along the lateral stripe, the cells along both stripes of each embryo were independently scored. Presented p-values derived from unpaired two-tailed t-test between WT (or heterozygous for the melanocyte counts) and the genotype corresponding to each row. DS, dorsal stripe; DT, dorsal trunk; H, head; LS, lateral stripe; MP, migration paths; VS, ventral stripe; VT, ventral trunk. (DOCX) [file pone.0244794.s005.docx]

**S1 Table: Additional information on the assessment of live embryonic phenotypes.**

| **Stage/assay** | **Genotype/scored anatomical region** | **No. of total scored embryos** | **Mean cell No.** | **Standard deviation** | **p-value** |
| --- | --- | --- | --- | --- | --- |
| 30 hpf/ melanocyte scoring | *tfec^+/+^* or *tfec^+/ba6^*/DT | 41 | 40 | 12 |  |
|  | *tfec^ba6/ba6^*/DT | 41 | 16 | 4 | 4.3 x 10^-10^ |
|  | *tfec^+/+^* or *tfec^+/ba6^*/MP | 19 | 16 | 10 |  |
|  | *tfec^ba6/ba6^*/MP | 19 | 6 | 3 | 8.9 x 10^-16^ |
| 4 dpf/ melanocyte scoring | *tfec^+/+^* or *tfec^+/ba6^*/DS | 44 | 78 | 7 |  |
|  | *tfec^ba6/ba6^*/DS | 38 | 81 | 9 | 0.10 |
|  | *tfec^+/+^* or *tfec^+/ba6^*/LS | 44 | 24 | 4 |  |
|  | *tfec^ba6/ba6^*/LS | 38 | 25 | 4 | 0.04 |
|  | *tfec^+/+^* or *tfec^+/ba6^*/VS | 20 | 35 | 3 |  |
|  | *tfec^ba6/ba6^*/VS | 10 | 38 | 4 | 0.11 |
|  | *tfec^+/+^* or *tfec^+/ba6^*/H | 44 | 37 | 4 |  |
|  | *tfec^ba6/ba6^*/H | 38 | 46 | 5 | 7.7 x 10^-12^ |
| 3 dpf/ iridophore scoring | *tfec^+/+^* /DS | 31 | 30 | 4 |  |
|  | *tfec^+/ba6^*/DS | 11 | 31 | 2 | 0.43 |
|  | *tfec^ba6/ba6^*/DS | 10 | 1 | 2 | 1.3 x 10^-28^ |
|  | *tfec^+/+^* /VS | 31 | 24 | 4 |  |
|  | *tfec^+/ba6^*/VS | 11 | 25 | 3 | 0.45 |
|  | *tfec^ba6/ba6^*/VS | 10 | 0 | 1 | 4.2 x 10^-28^ |
