# Supplementary material for: The MITF paralog tfec is required in neural crest development for fate specification of the iridophore lineage from a multipotent pigment cell progenitor
Source: PLoS One. 2021 Jan 13;16(1):e0244794. doi: 10.1371/journal.pone.0244794 (PMC7806166; doi:10.1371/journal.pone.0244794)
Supplement: S2 Table — The Pearson’s chi-squared test for goodness of fit is used to calculate the likelihood of a non-WT phenotype, which is consistently present in a number of embryos (1st sub-column of each of the 4 developmental stages) within a batch of WT, heterozygous and homozygous mutant siblings, correlating with homozygosity of the mutant allele in those individuals. Due to the recessive nature of investigated alleles, 25% of embryos in each batch are expected to be homozygous mutants. Therefore, the p-value derived from the chi-squared test indicates whether the number of individuals with an alternative phenotype conform to the expected 25% (null hypothesis), with any deviation being attributable to random chance (p > 0.1), or whether the numbers deviate significantly from the expected (p ≤ 0.1; null hypothesis is rejected). Where observed phenotypes did not significantly correlate with expected Mendelian ratios, i.e. where there is unlikely to be a mutant phenotype, the corresponding counts and p-values are red and bold. For the data on sox10t3/t3; sox9bfh313/fh313 double mutants, the orange cells indicate how many embryos in the sample showed the known sox9bfh313/fh313 phenotype, the green cells indicate number of embryos with the sox10t3/t3 phenotype and blue cells indicate unexpected alternative phenotypes, likely owed to double loss of function. (DOCX) [file pone.0244794.s006.docx]

**S2 Table: Statistics of loss of function experiments.**

| **WISH pattern** | **Genotype** | **Stage** | | | | | | | |
| --- | --- | --- | --- | --- | --- | --- | --- | --- | --- |
|  |  | 18 hpf | | 24 hpf | | 30 hpf | | 36 hpf | |
| *sox10* | *tfec^ba6/ba6^* | **0 of 54** | **p < 0.001** | **0 of 48** | **p < 0.001** | **0 of 22** | **p < 0.001** |  |  |
| *pnp4a* | *tfec^ba6/ba6^* |  |  | 11 of 49 | p ≈ 0.7 | 12 of 48 | p ≈ 0.95 |  |  |
| *ltk* | *tfec^ba6/ba6^* |  |  | 12 of 49 | p ≈ 0.95 |  |  |  |  |
| *mitfa* | *tfec^ba6/ba6^* |  |  | 10 of 50 | 0.3 < p < 0.5 | 7 of 35 | p ≈ 0.5 |  |  |
| *tfec* | *tfec^ba6/ba6^* |  |  | 14 of 48 | p ≈ 0.5 |  |  | 14 of 59 | 0.8 < p < 0.9 |
|  | *sox10^t3/t3^* | **0 of 41** | **p < 0.001** | 20 of 74 | 0.6 < p < 0.7 | 17 of 66 | p ≈ 0.9 | 15 of 51 | 0.4 < p < 0.5 |
|  | *mitfa^w2/w2^* | **0 of 48** | **p < 0.001** | 21 of 62 | 0.1 < p < 0.2 | 13 of 39 | 0.2 < p < 0.3 |  |  |
|  | *foxd3^sa20726/sa20726^* | **0 of 31** | **p < 0.001** | 9 of 57 | 0.1 < p < 0.2 | 17 of 52 | 0.2 < p < 0.3 |  |  |
|  | *sox9b^fh313/fh313^* | 11 of 45 | p ≈ 0.95 | 11 of 50 | 0.5 < p < 0.7 | 8 of 30 | 0.8 < p < 0.9 |  |  |
|  | *tfap2a^ts213/ ts213^* | **0 of 56** | **p < 0.001** | 14 of 45 | 0.3 < p < 0.5 |  |  |  |  |
|  | *sox10^t3/t3^*  *sox9b^fh313/fh313^* | 2 of 15 | p ≈ 0.7 | 11 of 55 | p ≈ 0.15 |  |  |  |  |
|  |  | **0 of 15** | **p ≈ 0.025** | 12 of 55 | 0.5 < p < 0.7 |  |  |  |  |
|  |  | **0 of 15** | **p ≈ 0.025** | 0 of 55 | **p < 0.001** |  |  |  |  |
